# EpimiR: a database of curated mutual regulation between miRNAs and epigenetic modifications

**DOI:** 10.1093/database/bau023

**Published:** 2014-03-28

**Authors:** Enyu Dai, Xuexin Yu, Yan Zhang, Fanlin Meng, Shuyuan Wang, Xinyi Liu, Dianming Liu, Jing Wang, Xia Li, Wei Jiang

**Affiliations:** College of Bioinformatics Science and Technology, Harbin Medical University, Harbin 150081, People's Republic of China

## Abstract

As two kinds of important gene expression regulators, both epigenetic modification and microRNA (miRNA) can play significant roles in a wide range of human diseases. Recently, many studies have demonstrated that epigenetics and miRNA can affect each other in various ways. In this study, we established the EpimiR database, which collects 1974 regulations between 19 kinds of epigenetic modifications (such as DNA methylation, histone acetylation, H3K4me3, H3S10p) and 617 miRNAs across seven species (including *Homo sapiens*, *Mus musculus*, *Rattus norvegicus*, *Gallus gallus*, Epstein–Barr virus, *Canis familiaris* and *Arabidopsis thaliana*) from >300 references in the literature. These regulations can be divided into two parts: miR2Epi (103 entries describing how miRNA regulates epigenetic modification) and Epi2miR (1871 entries describing how epigenetic modification affects miRNA). Each entry of EpimiR not only contains basic descriptions of the validated experiment (method, species, reference and so on) but also clearly illuminates the regulatory pathway between epigenetics and miRNA. As a supplement to the curated information, the EpimiR extends to gather predicted epigenetic features (such as predicted transcription start site, upstream CpG island) associated with miRNA for users to guide their future biological experiments. Finally, EpimiR offers download and submission pages. Thus, EpimiR provides a fairly comprehensive repository about the mutual regulation between epigenetic modifications and miRNAs, which will promote the research on the regulatory mechanism of epigenetics and miRNA.

**Database URL:** http://bioinfo.hrbmu.edu.cn/EpimiR/.

## Introduction

Epigenetics refers to the heritable changes in gene expression without a change in the DNA sequence itself, including DNA methylation and post-translational modifications of chromatin proteins (1). Under the catalysis of de novo DNA methyltransferases (DNMT3A and DNMT3B) and being maintained by DNA methyltransferase DNMT1, methyl groups are deposited on CpG cytosine residues, which participate in gene imprinting, X chromosome inactivation and control of cell type-specific gene expression patterns (2, 3). Furthermore, there exist a number of post-translational modifications (such as acetylation, methylation, phosphorylation and ubiquitinoylation) on histones that act as positive or negative epigenetic regulators of gene expression (4, 5).

MicroRNA (miRNA) is a class of small noncoding RNA molecules that usually lead to translational repression and gene silencing through the specifically binding 3′ untranslated region of messenger RNAs based on sequence complementation at the post-transcriptional level (6). Primary transcripts of miRNA genes can be up to several kilobases long and possess a 5′ 7-methylguanosine cap and a 3′ polyadenylated tail, implying transcription by RNA polymerse. Mature miRNAs are processed from hairpin precursors, which can either be encoded by dedicated miRNA genes that are often found in intergenic regions, or reside in the introns of protein-coding genes and be processed following transcription of the host genes (7, 8). Similar to coding gene, transcription of miRNA suffers from epigenetic control as well.

Recent evidence suggested that epigenetic control is involved in the regulation of miRNA gene expression in diverse ways. DNA methylation of promoter-associated CpG dinucleotides (especially in CpG islands) usually correlates with reduced transcription levels of corresponding miRNAs and subsequently induces the expression of miRNA target genes (9–11). Histone modifications have also been discovered to play positive or negative roles in controlling miRNA expression in multiple normal cells and diseases (12, 13). In addition, miRNA can be regulated by the altered epigenetic modification levels of miRNA biogenesis-related genes. Shibata’s group detected that trimethylation of histone H3 lysine 27 in the promoters of Dgcr8, P68, P72, Dicer, Ago3, Ago4 and Piwil4 could decrease these genes’ own expression and subsequently affect biogenesis of miRNAs in non-HBV genome-integrated hepatocellular carcinoma cell lines (14). Especially, epigenetic drugs, such as DNA methyltransferase inhibitor and histone deacetylase inhibitor, have been demonstrated to be able to result in the upregulation of miRNAs and provide a new idea for clinical treatment (15, 16).

On the other side of the coin, miRNA plays an important role in control of DNA methylation or histone modification through directly targeting epigenetic enzymes or functional protein complexes (17). For instance, miR-29b has been approved to induce global DNA hypomethylation and reactivate tumor suppressor gene P15 and ESR1 in acute myeloid leukemia by targeting DNMT3A and DNMT3B directly and DNMT1 indirectly (18). In addition, Yuan *et al*. (19) determined that miR-200a could upregulate global histone H3 acetylation levels through directly targeting the 3′ untranslated region of the HDAC4 messenger RNA and repressing expression of HDAC4 in hepatocellular carcinoma. Excitingly, the histone H3 acetylation levels at the promoter of miR-200a itself were also increased through an Sp1-dependent pathway that consequently activates its own transcription. Generally speaking, epigenetic modifications and miRNAs could construct a mutual regulation network to contribute to diseases.

Recently, many studies have revealed that epigenetics and miRNA can affect each other in many biological processes. Thus, a complete database that stores the experimentally validated interactions between epigenetic modifications and miRNAs is valuable to understand the molecular mechanisms of diseases and promote the targeted research toward epigenetics- or miRNA-related drugs. However, the relevant information is scattered in the massive body of literature, which is inconvenient for researchers to explore their mutual regulations. Accordingly, we developed a manually curated resource entitled ‘EpimiR’ (http://bioinfo.hrbmu.edu.cn/EpimiR/), which provided a comprehensive repertory of experimentally validated interactions between epigenetic modifications and miRNAs across multiple species and filled the vacancy in this field.

## Results

**Figure 1.**
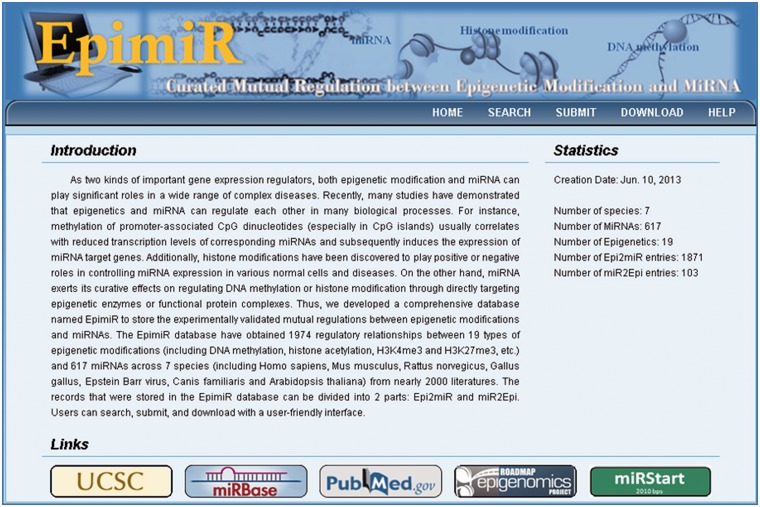
A snapshot of the graphical user interface to the database.

EpimiR provides a user-friendly interface to query detailed information on each experimentally validated interaction between epigenetics and miRNA documented in the database. A snapshot of the graphical user interface to the database is provided in Figure 1. In the ‘MiRNA Regulates Epigenetic Modification’ section, users can separately or synergistically select interested miRNA and epigenetic modifications to find out which epigenetic modifications could be regulated by the miRNA or which miRNAs could affect the epigenetics. Similarly, users can obtain which epigenetic modifications could influence the interested miRNA or which miRNAs could be in the control of the queried epigenetics in the ‘Epigenetic Modification Affects MiRNA Expression’ section, which supports fuzzy searching. As a supplement, if users cannot find experimentally validated interactions associated with their interested miRNAs in the miR2Epi or Epi2miR section, they can also search in the ‘Epigenetic Features for miRNA’ section to make use of the predicted collections to guide further experiments.

Before searching, users should choose to view a high-confidence-level or low-confidence-level record or both. Each curated entry in the EpimiR has been graded, which is more efficient for users to understand and use these records. High-confidence-level records usually provide sufficient evidence to present the regulatory process between miRNA and epigenetics. For low-confidence-level records, most of them merely demonstrate the existence of potential relationship between miRNAs and epigenetic modifications (details in Supplementary Material).

The ‘Result’ page lists all possible records that are related to users’ inputted queries and provides a download option for exporting search results. Each record offers a series of concise terms to exhibit the regulatory process. Moreover, users can retrieve more detailed information about the interested record in the ‘Detail’ page through clicking the ‘more … ’ hyperlink. Especially, EpimiR modeled all experimentally validated regulations as the miR2Epi pathway or Epi2miR pathway, which presents components of the pathway in bold in the ‘Detail’ page (Table 1).

**Table 1.** Definition and example of miR2Epi and Epi2miR pathway

Category	Definition		Example
**miR2Epi regulatory pathway**	**miR2Epi pathway** is the regulation wherein miRNA regulates epigenetic modification of ***Epi-Target*** through directly targeting ***Epi-Regulator***.	***Epi-Regulator*** is defined as the regulator of epigenetics.	In the discovery of Pospisil,V. *et al.* the miR2Epi pathway can be described as the regulatory process in which miR-29a regulates DNA methylation of FHIT and WWOX through directly targeting DNMT-3a and DNMT-3b (two important DNA methyltransferases). In this case, DNMT-3a and DNMT-3b are two Epi-Regulators, and FHIT and WWOX are corresponding to Epi-Targets. (PubMed ID: 21897363) (20)
		***Epi-Target*** indicates molecule whose expression is controlled by epigenetics.	
**Epi2miR regulatory pathway**	**Epi2miR pathway** is described as the process in which under the control of ***Epi-Regulator***, epigenetics regulates miRNA and then affects ***miR-Target***.	***Epi-Regulator*** is defined as the regulator of epigenetics.	In the discovery of Yamagishi *et al.*, the Epi2miR pathway can be described as the regulatory process wherein under the control of EZH2 and SUZ12 (two important components of polycomb repressive complex), H3K9me and H3K27me histone modification regulates miR-31 and then affects NIK. In this case, EZH2 and SUZ12 are two Epi-Regulators, and NIK is corresponding to miR-Target. (PubMed ID: 22264793)(21)
		***miR-Target*** is the gene that is approved to be directly targeted by miRNAs.	

Finally, EpimiR provides a submission page allowing other researchers to submit important interactions between epigenetics and miRNA that are not documented. Once approved by the submission review committee, the submitted record will be included in the database and made available to the public in the coming release. Moreover, users can acquire all curated records for systematic research in the download page.

### Case study

**Figure 2.**
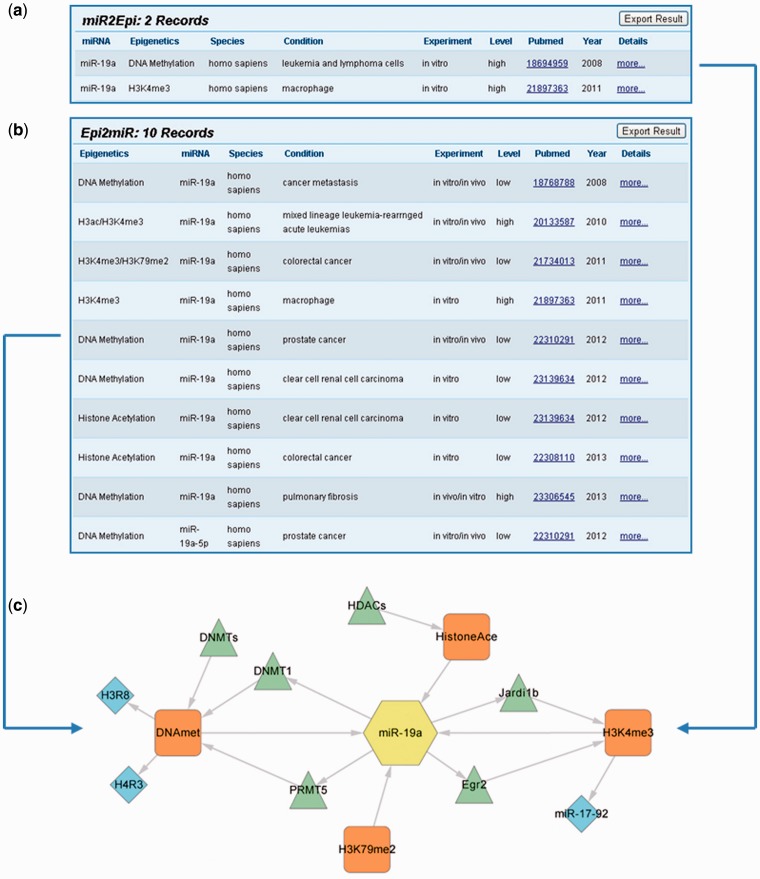
Searching result and integrated network of ‘miR-19a’.

It is worthwhile to note that epigenetic modifications and miRNAs could construct a mutual regulation network with EpimiR. Taking miR-19a as an example, we learned that miR-19a can both affect H3K4me3 states in human macrophage and control DNA methylation modification of human leukemia and lymphoma cells through querying in the ‘MiRNA Regulates Epigenetic Modifications’ section (Figure 2a). Accessing the detailed page, we further acquired that miR-19a regulates H3K4me3 states of the miR-17-92 cluster through directly targeting Egr2 and Jardi1b and influences DNA methylation of H3R8 and H4R3 by repressing PRMT5. Similarly, the epigenetic modifications that can affect miR-19a could be searched in the ‘Epigenetic Modification Affects miRNA Expression’ section. The searching result page shows nine records indicating that H3K4me3, H3K79me2, histone acetylation and DNA methylation can all affect miR-19a expression in diverse conditions (Figure 2b). To this end, we merged the aforementioned regulatory information and created a molecular network to reflect the interaction between miR-19a and diverse epigenetics (Figure 2c). The integrated network is valuable for systematically analyzing the synergistic roles of epigenetic modification and miRNA in diverse biological conditions or diseases.

## Discussion

The established resource not only provides an important reference for molecular biologists to understand and explain the regulatory mechanisms of miRNA and epigenetic modification in multiple types of biological processes but also offers potential epigenetic-associated biomarkers for predicting, diagnosing and treating diseases. Furthermore, the collections of EpimiR are valuable for system biologists to consider the role of the interactions between distinct regulatory levels in human diseases.

As stated in the aforementioned section, the epigenetics–miRNA relationships stored in the current release were collected through searching the PubMed database with a list of keywords. Although we gathered 1974 regulations from >300 literature citations, such a mechanism suffers from the lack of systematicness and comprehensiveness. By constantly updating, EpimiR will become a more comprehensive and user-friendly database about mutual regulations between epigenetics and miRNA from high-throughput method identification and low-throughput experiment validation.

## Materials and methods

Initial entries describing the regulatory relationships between epigenetic modification and miRNA are collected manually. We searched the PubMed database (22) with a list of keywords, such as ‘microRNA and epigenetics’, ‘miRNA and epigenetics’, ‘microRNA and DNA methylation’ and ‘microRNA and histone modification’. Through manual curation from ∼2000 literature references, we have obtained 1974 regulatory relationships between 19 kinds of epigenetic modifications (DNA methylation, histone acetylation, H3K4me3, H3S10p, etc.) and 617 miRNAs across seven species (including *Homo sapiens*, *Mus musculus*, *Rattus norvegicus*, *Gallus gallus*, Epstein–Barr virus, *Canis familiaris* and *Arabidopsis thaliana*) from 309 papers. These regulations can be compartmentalized into two parts: 103 miR2Epi records and 1871 Epi2miR records. Each entry of Epi2miR contains the detailed information about epigenetics that can regulate the expression of miRNAs, such as epigenetic modifications that impact miRNA expression, regulators that affect epigenetic modification, genes that are proven to be directly targeted by miRNAs, tissues or conditions for detection, experimental method, species and PubMed identifier (ID). Similarly, for the miR2Epi entry, the EpimiR database clearly elucidates the process of miRNA regulating epigenetics through listing the regulators of epigenetics that are directly targeted by miRNAs, tissues or conditions for detection, experimental method, species, PubMed ID, etc. Additionally, we extend to gather predicted miRNA genomic features associated with epigenetic modification. [such as type of miRNA locating area, position of transcription start site (23), upstream CpG island location (24) and target genes related to epigenetics (25)] associated with miRNA to complement to the curated information. Finally, EpimiR was developed using JSP, Tomcat 6.0.33 and MySQL 5.0 and runs under the Cent OS 5.5 system.

## Supplementary Data

Supplementary data are available at *Database* Online.

## Funding

Funds for Creative Research Groups of the National Natural Science Foundation of China (81121003), the National Natural Science Foundation of China (30900837) and the Foundation for University Key Teacher of the Education Department of Heilongjiang Province (1252G037). Funding for open access charge: The Foundation for University Key Teacher of the Education Department of Heilongjiang Province (1252G037).

*Conflict of interest*. None declared.
